# Comparative Clinical Study of Retention in Complete Denture Bases Using Green Stick Compound and Polycaprolactone Border Molding Materials: An In Vivo Study

**DOI:** 10.7759/cureus.110256

**Published:** 2026-06-04

**Authors:** Marwa Mohd Vazeer, Sreeramulu Basapogu, Pilli Bhavana, Divya Puli, Akhil Khadkekar, Shalini Karnam

**Affiliations:** 1 Prosthodontics, Government Dental College and Hospital, Hyderabad, IND

**Keywords:** border molding, denture retention, green stick compound, polycaprolactone, visual analogue scale

## Abstract

Background

Retention is a critical determinant of complete denture success, with border molding playing a pivotal role in establishing an effective peripheral seal. While green stick compound has long been the conventional material, polycaprolactone (PCL) has recently emerged as a promising alternative.

Aim

This study aimed to compare the retention, patient comfort, and operator handling characteristics of complete denture bases fabricated using green stick compound and PCL border molding materials.

Objectives

The objectives of this study were to assess the retention achieved with green stick compound, to evaluate the retention obtained using PCL, to compare patient comfort during border molding procedures with both materials, and to statistically analyze the differences in retention between the two groups.

Materials and methods

A comparative clinical study was conducted on 17 edentulous patients. Two custom trays were fabricated per patient, with border molding performed using green stick compound (Group A) and PCL (Group B). Final impressions were made with zinc oxide eugenol paste. Retention was objectively measured using a standardized pull test, patient comfort was assessed using the Visual Analogue Scale (VAS), and operator feedback was recorded via a study-specific structured Likert scale questionnaire.

Results

PCL demonstrated significantly higher retention (32.82 ± 12.59 N) compared to green stick compound (28.99 ± 10.32 N) (paired t-test; t = -4.395, p < 0.001). Patient comfort scores were significantly greater with PCL (9.41 vs. 8.65; Z = -3.557, p < 0.001). Operator feedback favored PCL, with significant differences observed in handling, adaptability, and reheating characteristics (p < 0.05). All denture bases in both groups were clinically stable during functional movements.

Conclusion

PCL provides superior denture retention, enhanced patient comfort, and improved handling characteristics compared to green stick compound. The improved retention may be attributed to its superior thermoplastic adaptability and ability to achieve an effective peripheral seal, making it a clinically advantageous alternative for border molding in complete denture fabrication.

## Introduction

Retention is widely regarded as a fundamental determinant of successful complete denture therapy, as it directly influences patient comfort, functional efficiency, and overall satisfaction [1]. Achieving optimal retention depends on several clinical procedures, among which border molding plays a pivotal role in establishing an effective peripheral seal [2]. Accurate border molding ensures proper flange extension, thereby enhancing denture retention and stability and reducing the likelihood of displacement during functional movements [3].

Green stick compound has traditionally been the material of choice for border molding due to its extensive clinical use and effectiveness in capturing the vestibular depth [4]. Despite these advantages, it is associated with certain limitations, including technique sensitivity and the need for repeated heating, which can make the procedure time-intensive and highly operator-dependent [5]; it requires direct flame heating, making it prone to overheating, distortion, and patient discomfort. Its repeated reheating cycles render the procedure highly operator-dependent. Polycaprolactone (PCL), on the other hand, eliminates the need for flame heating and offers improved patient comfort, but it still demands careful temperature control and multiple reheating steps to maintain workability. Thus, although PCL overcomes certain limitations associated with green stick compound, both materials remain technique-sensitive procedures that rely considerably on operator skill and experience.

A growing body of literature has explored the clinical relevance of PCL in prosthodontics. Shyani et al. (2019) introduced a single-step border molding technique utilizing this material, highlighting its favorable handling properties and patient acceptance compared to conventional approaches [6]. Yarapatineni et al. (2013) emphasized the critical influence of border molding on denture retention, noting that material selection can significantly affect treatment outcomes [7]. Likewise, Pachar et al. (2019) compared green stick compound, silicone putty, and polyether, demonstrating variability in retention performance among these materials [8]. More recent reviews have underscored the need for evidence-based selection of border molding materials, while also pointing out the absence of a clear consensus regarding newer options such as polycaprolactone [6,9].

Given the limited availability of direct comparative clinical evidence, the present study was designed to evaluate and contrast the retention of complete denture bases fabricated using green stick compound and PCL. By combining objective measurements of retention with assessments of patient comfort and operator experience, this investigation seeks to inform clinical decision-making and support the selection of materials that enhance both denture performance and patient satisfaction.

## Materials and methods

Study design

A comparative clinical study was conducted to evaluate denture base retention using two different border molding materials: green stick compound and PCL.

Sample size calculation

Sample size calculation was performed using G*Power software (version 3.9.1, Heinrich Heine University Düsseldorf, Germany); the required sample size was determined using a priori power analysis for a paired t-test. A two‑tailed test was performed with an effect size of dz = 0.741, a significance level of 0.05, and a desired power of 0.80. The analysis yielded a noncentrality parameter of 3.055, a critical t-value of 2.119, and degrees of freedom (df) = 16. Based on these calculations, the total sample size required was 17 participants. This ensured adequate statistical power to detect clinically significant differences between the two materials.

Sample selection

Seventeen patients, completely edentulous or maxillary edentulous, who reported to the Department of Prosthodontics, Government Dental College and Hospital, Hyderabad, India, were selected for the study. The inclusion of both completely edentulous and maxillary edentulous patients was intended to improve the clinical applicability of the findings to a broader patient population commonly encountered in prosthodontic practice.

Inclusion Criteria

Patients included in the study demonstrated well‑formed ridges with adequate height and contour, providing a favorable foundation for denture fabrication and retention. All participants were in good systemic health, without systemic diseases or conditions such as uncontrolled diabetes, autoimmune disorders, or mucosal‑affecting syndromes that could compromise oral mucosa integrity or healing. In addition, only cooperative and motivated individuals who were willing to adhere to instructions and attend follow‑up visits were selected.

Exclusion Criteria

Patients with severely resorbed ridges, where advanced ridge resorption resulted in poor anatomical support and compromised denture stability, were excluded from the study. Individuals presenting with mucosal pathology, including oral lesions, inflammatory conditions, or other abnormalities that could interfere with impression making or denture adaptation, were also not considered. Patients with neuromuscular incoordination, such as tremors or impaired tongue and cheek control that could hinder border molding or denture retention, were excluded. In addition, individuals with systemic contraindications, systemic conditions negatively affecting oral tissues or healing capacity, were deemed unsuitable for inclusion.

Ethical considerations

The study protocol was approved by the Institutional Review Board of the institution (Ref. No. IEC/OMC/M.NO (17)/P‑186). Written informed consent was obtained from all patients prior to participation to use their records for study purposes. 

Procedure

For each patient, the primary impression of the maxillary arch was made using an irreversible hydrocolloid impression material (Tropicalgin, Zhermack, S.p.A., Badia Polesine, Italy) in a suitably sized perforated stock tray. The impressions were immediately poured in Type II dental plaster (Kalstone, Kalabhai Pvt. Ltd., Mumbai, India) to obtain the primary cast.

**Figure 1:**
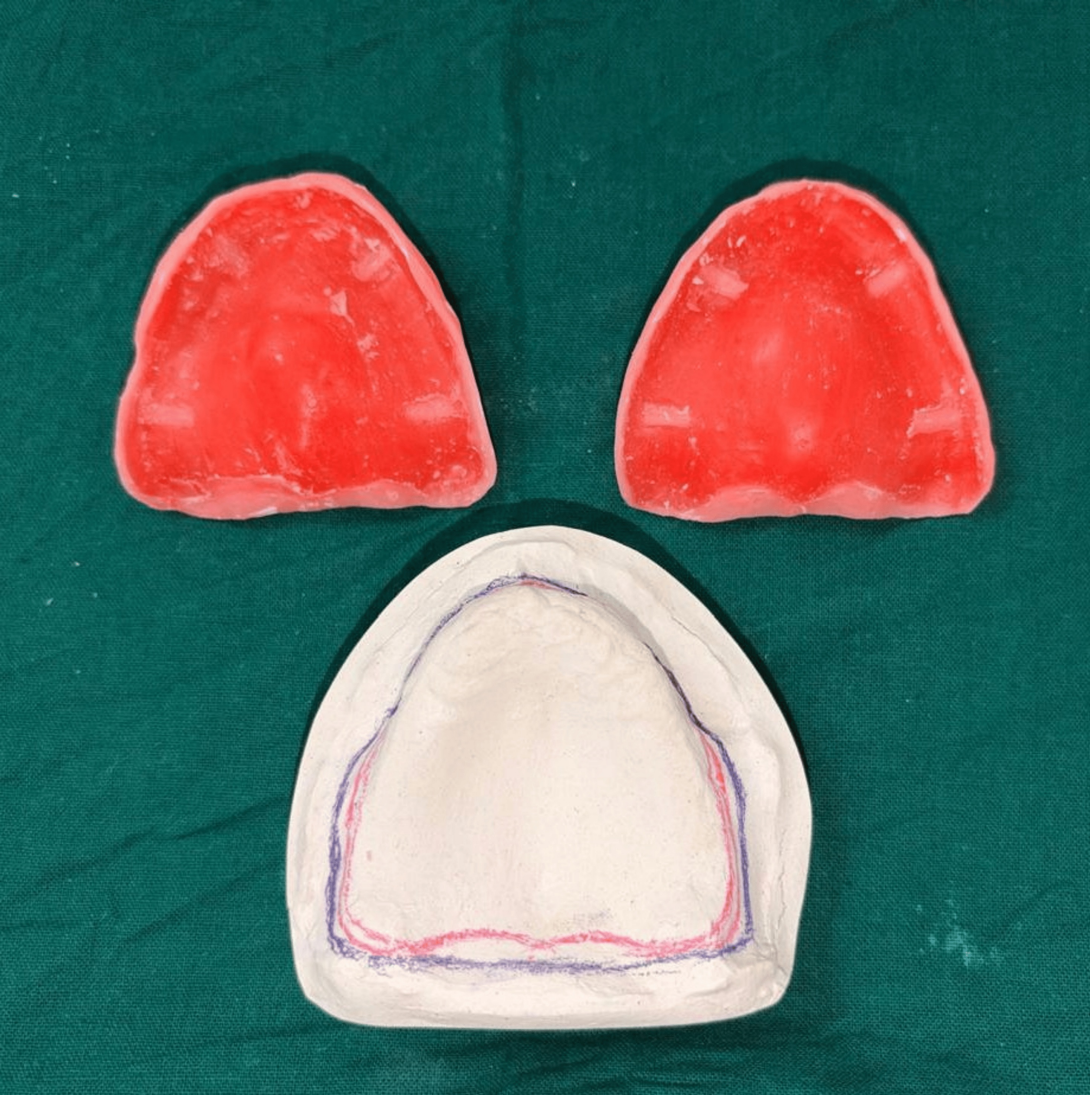
Custom trays fabricated for border molding. Two custom trays were fabricated on the same primary cast for the border molding procedure using two different materials: green stick compound and polycaprolactone.

The casts were outlined 2 mm short of the vestibule and relieved for the fabrication of custom impression trays using autopolymerizing acrylic resin (Dental Products of India, RR Cold Cure, The Bombay Burmah Trading Corporation Ltd., Mumbai, India). Two identical custom trays were fabricated for each patient to allow border molding with both test materials under standardized conditions. Each tray was constructed with a uniform 2 mm wax spacer to provide adequate relief for impression material, except in the posterior palatal seal area, where the spacer was intentionally omitted to ensure proper tissue contact and retention. Tissue stops were incorporated in the canine and molar regions to stabilize the tray and maintain consistent spacer thickness during impression making (Figure 1).

Each tray was placed intraorally, and the peripheries were trimmed so that they were 2-3 mm short of the tissue reflection, except in the hamular notch and posterior palatal seal area, in accordance with established prosthodontic protocols [1,2].

Border molding was performed for each group using the following materials: Group A: green stick compound, the conventional material widely used in clinical practice; Group B: PCL, a newer thermoplastic material introduced for simplified border molding.

For Group A, one of the custom trays was used to perform border molding with green stick compound (Dental Products of India, Pinnacle, The Bombay Burmah Trading Corporation Ltd., Mumbai, India). Border molding was performed by applying green stick compound incrementally to the buccal and labial tray borders, with lips and cheeks manipulated to capture functional movements. For the posterior palatal seal, a softened compound was adapted while patients performed the Valsalva maneuver to record the posterior palatal seal region functionally.

**Figure 2:**
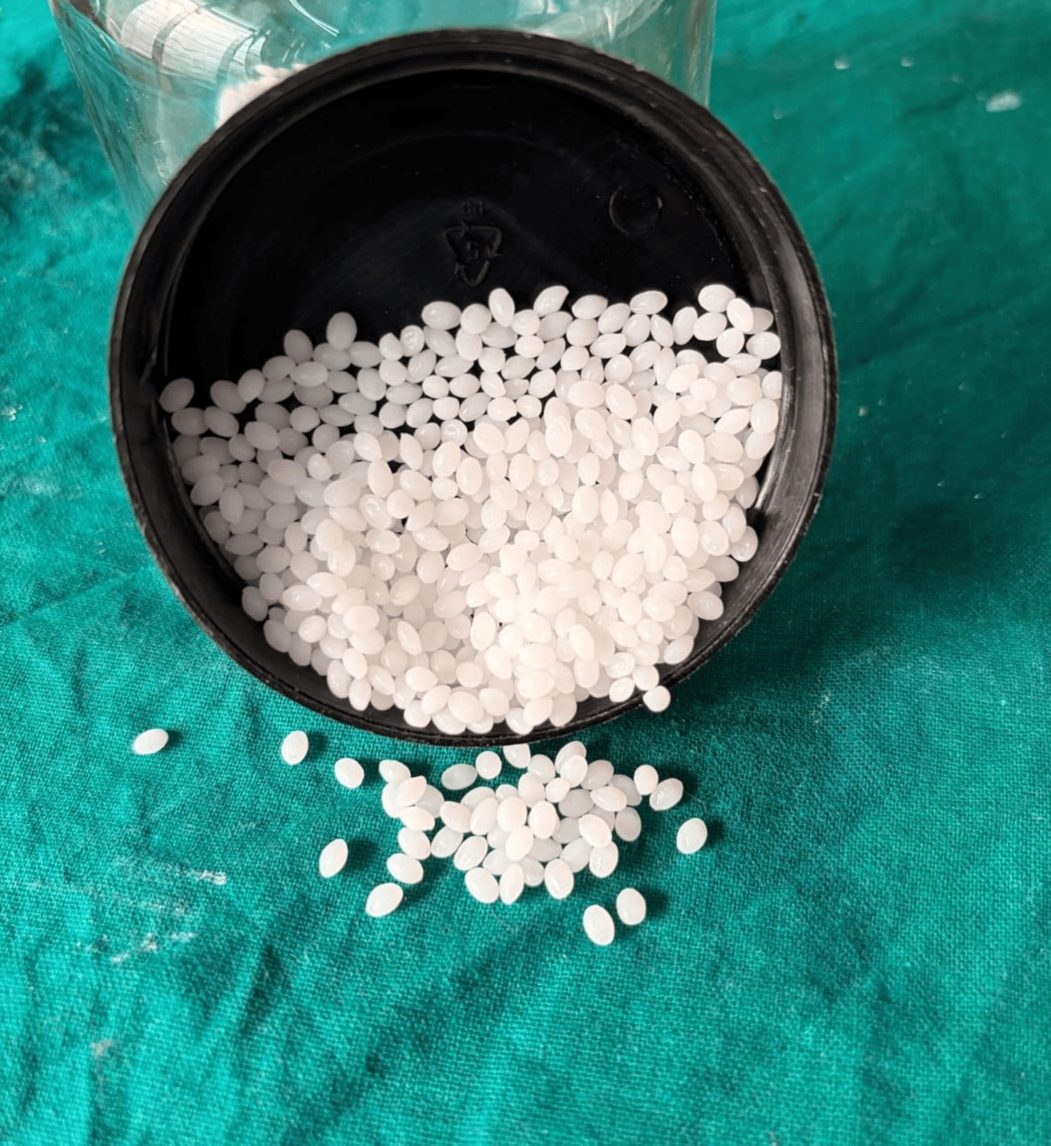
Polycaprolactone beads

For Group B, the second custom tray was used to perform border molding with PCL thermoplastic material (PCL-CL1005CS, Nomisma Healthcare Pvt. Ltd., Vadodara, India) (Figure 2).

The procedure was carried out using the single-step technique described by Shyani et al. (2019) [6].

Material Preparation

PCL beads were immersed in hot water maintained at approximately 60 °C until they became soft and pliable. Once adequately softened, the material was carefully removed from the water and blotted dry to eliminate excess moisture. The pliable beads were then adapted uniformly along the borders of the custom tray, ensuring even distribution and intimate contact with the tray periphery. Care was taken to press and contour the material against the functional depth of the vestibule, allowing accurate recording of border extensions, and reheating the beads as necessary to maintain workability and avoid premature hardening.

**Figure 3:**
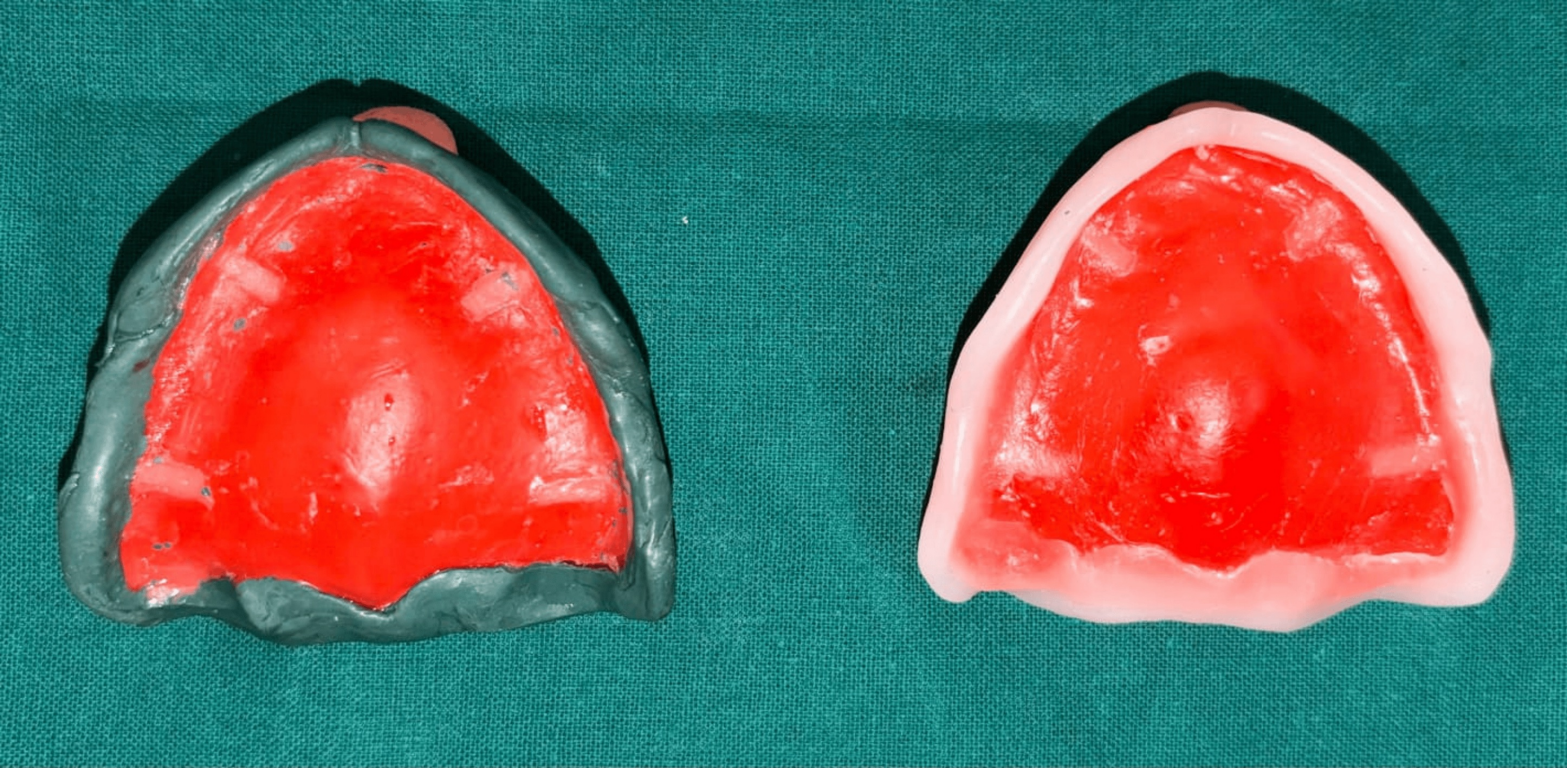
Maxillary custom trays border molded with green stick compound and polycaprolactone.

Functional molding was carried out by inserting the tray intra-orally and manipulating the lips and cheeks in multiple directions to record the buccal and labial vestibular movements. The posterior palatal seal was molded by instructing patients to perform functional movements, including the Valsalva maneuver, ensuring accurate registration of the posterior palatal tissues (Figure 3).

Excess green stick compound and PCL extending onto the ridge were carefully trimmed with a Bard‑Parker blade no. 15 to ensure proper tray adaptation. Following removal of the wax spacer, relief holes were created over the median palatal raphe and lateral regions of the hard palate to provide tissue relief and enhance retention of the impression material.

**Figure 4:**
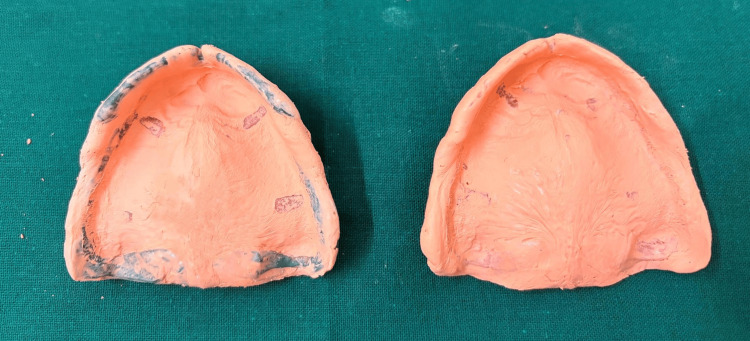
Border-molded custom trays with secondary impressions. Maxillary custom trays border molded with green stick compound and polycaprolactone, carrying secondary impressions made with zinc oxide eugenol impression paste for final impression recording.

Final impressions were made using zinc oxide eugenol (Dental Products of India, ZOE impression paste, Mumbai, India) impression paste; the trays were loaded with ZOE and inserted intra-orally, ensuring uniform seating and border adaptation. Patients were instructed to perform functional movements to record the dynamic extensions of the vestibular tissues (Figure 4). The completed impressions were poured in type III dental stone (Kalstone, Kalabhai Pvt. Ltd., Mumbai, Maharashtra, India) to obtain the definitive casts, which were subsequently used for the fabrication of heat-cured acrylic resin denture bases.

Border molding procedures were performed by three operators. For each participant, the same operator performed both Group A and Group B procedures to minimize inter-operator variability.

Assessment methods

Evaluation of the retention and stability provided by the test materials was carried out using both objective and subjective assessment protocols.

Objective Assessment

**Figure 5:**
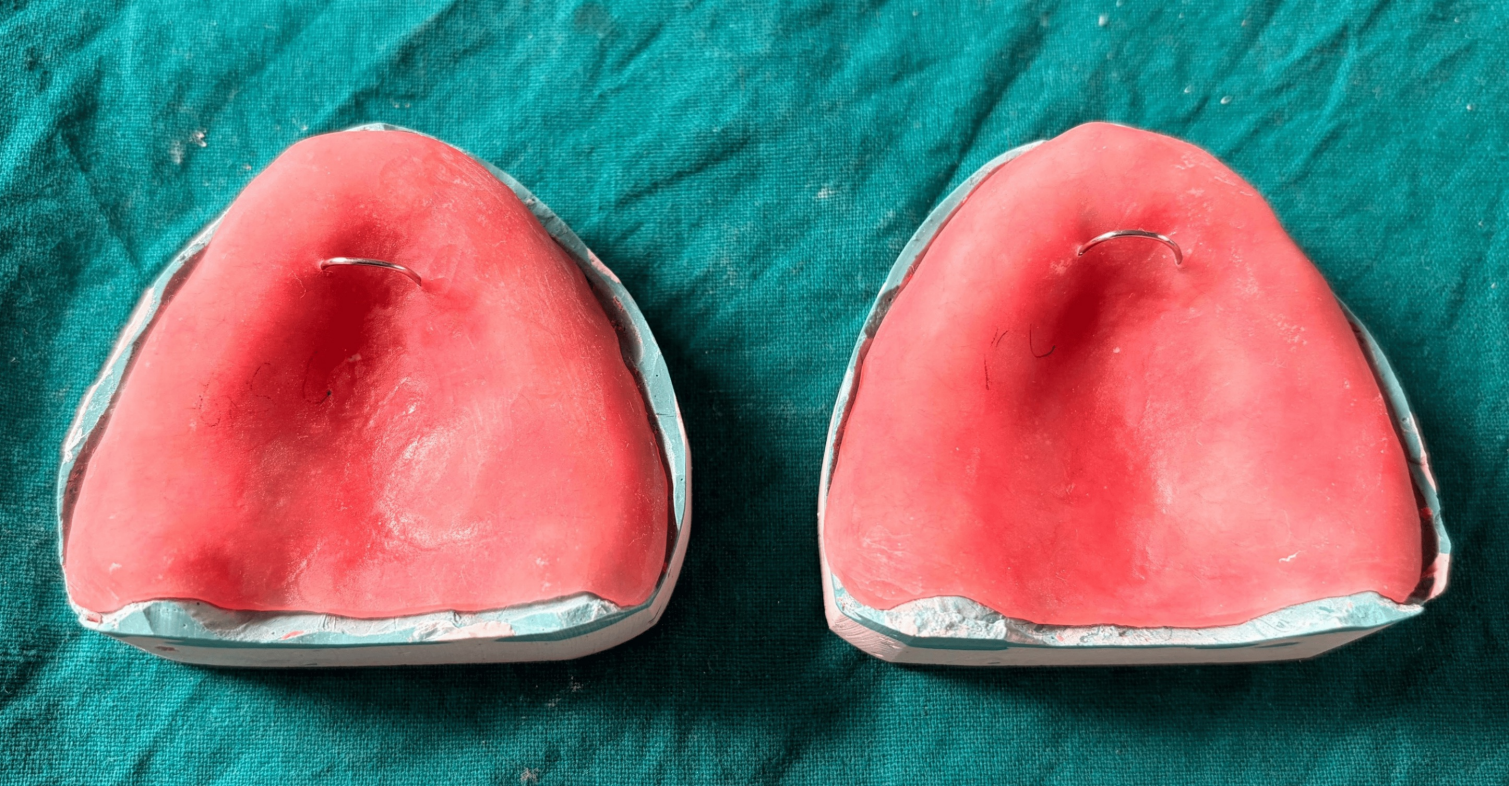
Maxillary denture bases fabricated for digital retention assessment. Heat-cured maxillary denture bases fabricated from custom trays border molded with green stick compound and polycaprolactone, incorporating anterior palatal 19-gauge stainless steel wire loops for attachment to the digital force gauge during retention assessment.

Retention was quantitatively measured by recording resistance to dislodgement with a standardized pull test described by Jacobson and Krol [5]. For this purpose, a 19‑gauge stainless steel wire hook was custom‑fabricated and securely affixed to the anterior palatal region of each denture base (Figure 5). This positioning ensured uniform distribution of applied forces and minimized distortion of the denture base during testing.

**Figure 6:**
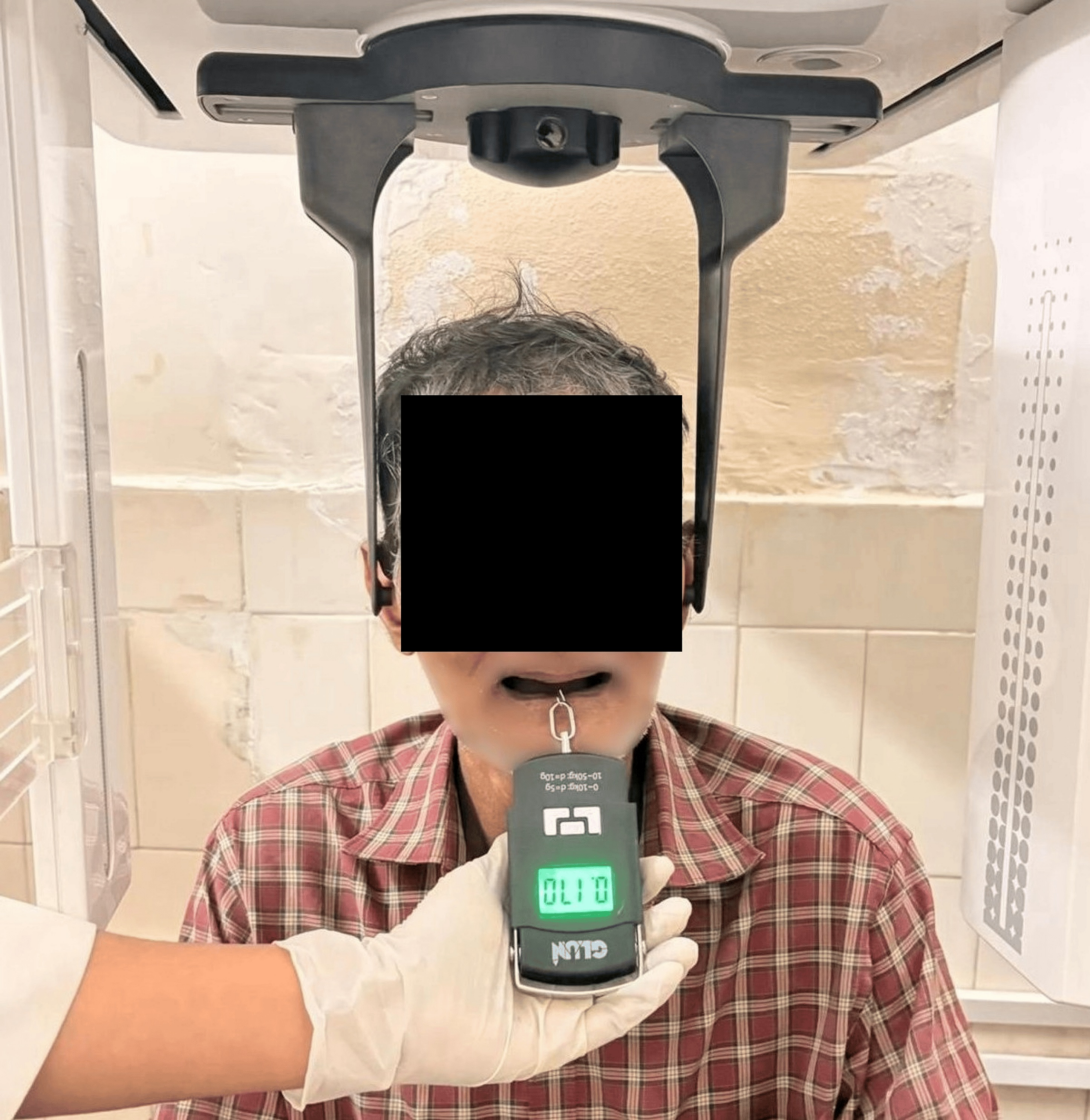
Clinical setup for digital measurement of denture base dislodgement force. Clinical photograph showing digital measurement of trial denture base dislodgement force using a digital force gauge with cephalostat-assisted head stabilization during retention assessment.

Controlled vertical dislodging forces were applied using a digital weighing device (Glun LCD Electronic Balance, Shenzhen City, China), which provided precise force measurements in Newtons (N). The procedure was performed under standardized conditions: the patient’s head was stabilized with a cephalostat (Figure 6) to eliminate variability in positioning and to maintain reproducibility across all cases.

The maximum force required to overcome the peripheral seal and dislodge the denture base was recorded as the retention value. To enhance reliability, each measurement was repeated three times per denture base, and the mean of these readings was calculated for statistical analysis to ensure consistent and reproducible outcomes.

Subjective Assessment

Patient comfort during border molding procedures was assessed using the Visual Analogue Scale (VAS), a validated subjective measurement tool [10]. Immediately following each procedure, patients rated their comfort level for both materials on a 10 cm line where “0 = Discomfort” and “10 = Maximum Comfort.” Scores for both green stick compound and PCL were recorded, and mean values with standard deviations were calculated for statistical comparison. Denture base stability was further evaluated clinically through functional movement tests, where patients performed routine oral actions such as mouth opening, speaking, swallowing, and tongue movements. Stability was judged based on maintenance of the peripheral seal and positional integrity, with outcomes recorded qualitatively as either stable or unstable.

In addition, operator feedback was obtained using a structured, study-specific Likert scale questionnaire (Appendix A). Prior to implementation, the questionnaire underwent content validation and was distributed among 10 experienced prosthodontists, who evaluated each item for clarity, relevance, understandability, and appropriateness of the assessed parameters. Items demonstrating acceptable content validity were retained for the final study.

Clinicians assessed each material across five parameters, and the responses were recorded on a 5‑point Likert scale (1 = strongly disagree, 5 = strongly agree). Mean scores with standard deviations were calculated for each parameter. All collected data were subjected to appropriate statistical analysis to determine the significance of observed differences.

## Results

The present study evaluated patient comfort, denture retention, and operator feedback for complete denture bases fabricated using green stick compound and PCL border molding materials. Data were analyzed using appropriate statistical tests: the Wilcoxon signed‑rank test for patient‑reported outcomes and operator feedback, the paired t‑test for retention forces, and the marginal homogeneity test for item‑wise operator ratings.

Analysis of patient-reported outcomes indicated that PCL produced significantly higher comfort scores on the VAS compared to green stick compound (mean 9.41 vs. 8.65; Wilcoxon signed rank test, Z = -3.557, p < 0.001), as presented in Table 1.

**Table 1:** Mean comparison of Visual Analogue Scale (VAS) scores between the groups. Group A: green stick compound, the conventional material widely used in clinical practice; Group B: PCL, a newer thermoplastic material introduced for simplified border molding.

Groups	n	Mean	SD	Test value	P-value
A	17	8.6471	0.49259	-3.557	<0.001*
B	17	9.4118	0.53722		

Objective assessment of retention using a force gauge likewise demonstrated improved performance for PCL (mean 32.82 ± 12.59 N) relative to green stick compound (mean 28.99 ± 10.32 N). This difference was statistically significant according to the paired t-test (t = -4.395, df = 16, p < 0.001), as shown in Table 2.

**Table 2:** Mean comparison of retention between the groups. Group A: green stick compound, the conventional material widely used in clinical practice; Group B: PCL, a newer thermoplastic material introduced for simplified border molding.

Groups	n	Mean	SD	Test value	P-value
A	17	28.9941	10.32076	-4.395	<0.001*
B	17	32.8176	12.58666		

Table 3 reported stability outcomes where all denture bases in both groups were stable during functional movements (100%), with no cases of instability observed. Hence, no statistical analysis was performed.

**Table 3:** Functional stability assessment of denture bases between the two groups. Group A: green stick compound, the conventional material widely used in clinical practice; Group B: PCL, a newer thermoplastic material introduced for simplified border molding.

Stability	Group A n (%)	Group B n (%)
Stable	17 (100)	17 (100)
Unstable	0 (0)	0 (0)

Operator evaluations supported these findings, with PCL receiving higher ratings across key parameters, including ease of handling, reheating capability, and adaptability (Table 4).

**Table 4:** Comparative operator assessment of test materials. Q: question; Q1-Q5 are listed in the operator feedback questionnaire in Appendix A. Group A: green stick compound, the conventional material widely used in clinical practice; Group B: PCL, a newer thermoplastic material introduced for simplified border molding.

Item	Group	Responses- Operators feedback										Test value	P-value
		1		2		3		4		5			
		n	%	n	%	n	%	n	%	n	%		
Q1	A	0	0	0	0	0	0	17	100	0	0	-	-
	B	0	0	0	0	0	0	17	100	0	0		
Q2	A	0	0	0	0	10	58.8	7	41.2	0	0	-3.812	<0.001*
	B	0	0	0	0	0	0	2	11.8	15	88.2		
Q3	A	0	0	0	0	0	0	17	100	0	0	1.030	1.000
	B	0	0	0	0	1	5.9	16	94.1	0	0		
Q4	A	0	0	8	47.1	8	47.1	1	5.9	0	0	-3.839	<0.001*
	B	0	0	0	0	0	0	3	17.6	14	82.4		
Q5	A	0	0	0	0	2	11.9	15	88.2	0	0	-3.000	0.003*
	B	0	0	0	0	0	0	7	41.2	10	58.8		

The findings presented in Table 4 indicate that no test statistic was computed for Question 1 (Q1), as both groups showed identical response distributions. For Q2, PCL received significantly higher ratings than the green stick compound (p < 0.001). In Q3, no significant difference was observed between the two materials (p = 1.000). In contrast, Q4 demonstrated that PCL was rated significantly higher (p < 0.001). Similarly, Q5 revealed that PCL was rated more positively (p = 0.003). Overall, feedback from operators favored PCL in most domains, except adaptation, where both materials performed similarly.

Statistical analysis using the marginal homogeneity test indicated significant differences in most assessed domains (p < 0.05). Furthermore, the overall operator feedback score (Table 5) was notably higher for PCL (22.24 ± 0.83) than for green stick compound (17.88 ± 0.78), with the difference confirmed as statistically significant by the Wilcoxon signed-rank test (Z = -3.676, p < 0.001).

**Table 5:** Mean comparision of operator feedback between the groups. Group A: green stick compound, the conventional material widely used in clinical practice; Group B: PCL, a newer thermoplastic material introduced for simplified border molding.

Groups	N	Mean	SD	Test value	P-value
A	17	17.8824	0.78121	-3.676	<0.001*
B	17	22.2353	0.83137		

Collectively, these results demonstrate that PCL significantly improved patient comfort, retention, and operator handling characteristics compared to green stick compound.

## Discussion

In this study, denture retention was the primary outcome measure, with patient comfort and operator feedback serving as complementary parameters to compare green stick compound and PCL for border molding procedures. The findings demonstrated that PCL achieved superior retention outcomes, along with significantly higher patient comfort scores and more favorable operator ratings. These results underscore the clinical advantage of PCL as a border-molding material, particularly in enhancing denture retention while also improving overall treatment experience.

Retention analysis revealed that PCL demonstrated superior performance compared to the green stick compound. The mean peak dislodging force recorded for PCL was 32.82 ± 12.59 N, significantly higher than that of the green stick compound (28.99 ± 10.32 N). Statistical comparison using the paired samples t‑test confirmed this difference to be highly significant (t = -4.395, p < 0.001). These findings indicate that PCL provided a more effective peripheral seal and greater resistance to dislodgement under standardized testing conditions. 

The enhanced retention observed with PCL may be attributed to its thermoplastic nature and superior flow characteristics, which allow better adaptation to the functional depth and width of the vestibular tissues during border molding. Compared to green stick compound, PCL exhibited greater flexibility and a prolonged working time after softening, thereby enabling more accurate recording of peripheral tissues during functional movements. Its ability to be reheated and readapted without significant distortion further facilitated incremental corrections, improved border accuracy, and enhanced denture stability. Collectively, these properties likely contributed to a more effective peripheral seal and consequently greater resistance to dislodgement.

Although the mean retention difference between the materials was approximately 3.8 N, even modest improvements in retention may contribute clinically to enhanced denture stability, improved resistance to displacement during function, and greater patient confidence during mastication and speech.

Patient comfort scores (mean = 9.41 ± 0.54 vs. 8.65 ± 0.49; Wilcoxon signed-rank test, Z = -3.557, p < 0.001) and operator ratings (mean = 22.24 ± 0.83 vs. 17.88 ± 0.78; Z = -3.676, p < 0.001) also significantly favored PCL.

Retention remains a critical determinant of denture success. In the present study, PCL was associated with improved retention outcomes. Previous investigations provide mixed evidence in this regard. Yarapatineni et al. (2013) [7] reported comparable retention between single-step silicone putty and sectional green stick compound, whereas Qanungo et al. (2016) [11] found superior retention with sectional green stick compound over condensation silicone. Kundu et al. (2026) [12] suggested that although green stick remains reliable, elastomeric materials such as polyether and condensation silicone can achieve satisfactory retention with reduced effort and time. Similarly, Tomer et al. (2023) [13] demonstrated significant differences in retention between green stick and condensation silicone, with higher values observed for silicone in denture base evaluations. Zarir et al. (2022) [14] further noted that the addition of silicone produces more accurate border morphology compared to the green stick compound. Collectively, these findings indicate that retention is strongly influenced by both material properties and clinical technique, supporting the present results that highlight the adaptability of PCL in achieving consistent peripheral seals.

Patient comfort, measured using the VAS, was significantly improved with PCL (Z = -3.557, p < 0.001). This method of evaluation is consistent with Chander (2019) [15], who established VAS as a dependable tool for assessing denture satisfaction, and with Ohara et al. (2020) [16], who reported enhanced comfort with digital denture techniques. The relatively low softening temperature of polycaprolactone may contribute to reduced intra-oral discomfort and a lower risk of thermal injury, thereby explaining the higher comfort scores.

Operator feedback, collected via a structured study-specific Likert scale questionnaire, consistently favored PCL across most domains. Significant differences were observed in ease of handling (p < 0.001), reheating (p < 0.001), and overall preference (p = 0.003). Punj (2017) [17] emphasized the importance of handling properties in impression materials, while Sullivan and Artino (2013) [18] validated Likert scales as effective tools for capturing subjective perceptions.

PCL has recently gained attention in dentistry for its biocompatibility and versatility. This study contributes new clinical evidence supporting its use in border molding, expanding its potential applications in routine practice.

Limitations

This study provides useful information on the comparative performance of PCL and green stick compound. However, the small sample size (n = 17) limits the generalizability of the findings. In addition, retention values may be influenced by variations in patient anatomy, denture base adaptation, and minor differences in clinical technique that could not be completely controlled. The inclusion of both completely edentulous and maxillary edentulous patients may have introduced some heterogeneity in clinical conditions. The study also involved three operators, and although the same operator performed both procedures for each participant to minimize inter-operator variability, operator-related bias could not be completely eliminated. Blinding during border molding procedures was not feasible because of the distinct handling characteristics of the materials. Furthermore, the operator questionnaire was study-specific, and although it underwent content validation by ten experienced prosthodontists prior to implementation, it was not a standardized or previously validated instrument. Retention was evaluated only for maxillary dentures, consistent with previous studies, and therefore, the findings may not be directly applicable to mandibular dentures. The short-term nature of the study limited the ability to evaluate long-term clinical performance and patient adaptation.

## Conclusions

Within the constraints of this study, PCL demonstrated clear advantages in patient comfort and operator handling when compared to green stick compound, while retention outcomes remained influenced by clinical technique. These findings suggest that thermoplastic materials such as PCL can enhance both patient experience and clinical efficiency in border molding procedures. Further large-scale, multi-operator investigations are necessary to confirm these results and support their adoption as a reliable alternative in routine prosthodontic practice.

## Appendices

Appendix A

Questionnaire Assessment for Operator Feedback

The clinician will record feedback on the handling properties of each material using a 5-point Likert scale (1= strongly disagree, 5= strongly agree).

1.  The material was easy to soften and manipulate

o   Strongly Disagree

o   Disagree

o   Neutral

o   Agree

o   Strongly Agree

2. The material adapted well to vestibular tissues

o   Strongly Disagree

o   Disagree

o   Neutral

o   Agree

o   Strongly Agree

3. The material allowed multiple reheating cycles without degradation

o    Strongly Disagree

o   Disagree

o   Neutral

o   Agree

o   Strongly Agree

4. The material was comfortable to use without risk of patient burns

o   Strongly Disagree

o   Disagree

o   Neutral

o   Agree

o   Strongly Agree

5. Overall, I prefer this material for border molding procedures

o   Strongly Disagree

o   Disagree

o   Neutral

o   Agree

o   Strongly Agree
